# A Semi-distributed Model for Predicting Faecal Coliform in Urban Stormwater by Integrating SWMM and MOPUS

**DOI:** 10.3390/ijerph16050847

**Published:** 2019-03-08

**Authors:** Xiaoshu Hou, Lei Chen, Jiali Qiu, Yali Zhang, Zhenyao Shen

**Affiliations:** 1 State Key Laboratory of Water Environment Simulation, School of Environment, Beijing Normal University, Beijing 100875, China; houxiaoshu_84@126.com (X.H.); chenlei1982bnu@bnu.edu.cn (L.C.); 2 Institute of Geographic Sciences and Natural Resources Research, Chinese Academy of Sciences, Beijing 100101, China; qjl@mail.bnu.edu.cn (J.Q.); zhangyali@igsnrr.ac.cn (Y.Z.)

**Keywords:** SWMM, MOPUS, semi-distributed, microorganism modelling, faecal coliform

## Abstract

The microbial contamination of urban stormwater has an important impact on human health and stormwater reuse. This study develops an exploratory semi-distributed model, MOPUS_S, which can simulate faecal coliform levels in separate sewer systems in urban catchments. The MOPUS_S was built by coupling the SWMM model and the microbial MOPUS model. The parameters associated with the deposition and wash-off of microorganisms were more influential than those related to microorganism survival processes. Compared to other existing bacterial models, MOPUS_S showed comparable performance in predicting faecal coliform concentrations. The performance varied largely between rainfall events, with Nash-Sutcliffe efficiency (NSE) values ranging from −5.03 to 0.39 and R^2^ ranging from −0.02 to 0.83, respectively. The model simulation results for low and medium concentrations were better than those for the peak concentrations. Poor simulation results of peak concentrations obviously affect the overall model performance. In general, MOPUS_S could be capable of predicting the faecal coliform load in urban catchments and be a useful tool for urban stormwater management planning.

## 1. Introduction

As a major source of urban non-point source (NPS) pollution, stormwater runoff contains high concentrations of biological pollutants that are directly related to disease outbreaks, aquatic biological toxicity and water quality degradation [1,2]. Of particular concern is contamination by microorganisms, especially pathogens, which are considered as a leading cause of impairment of rivers, streams, and estuaries around the world [3,4]. Microbial pathogens, including *Giardia*, *Cryptosporidium*, *Salmonella*, *Campylobacter*, and human viruses, have been found in urban stormwater [5,6,7,8]. Therefore, investigating microorganisms in urban stormwater is very necessary for water quality management and stormwater reuse.

However, the study of microorganisms in urban stormwater faces several challenges. For instance, it is really hard to accurately obtain the monitoring data of microorganisms due to high uncertainty related to stormwater sampling and analysis. Moreover, microorganisms are not only involved in the building-up and washing-off of pollutants at the surface and in the subsurface but also influenced by many factors (such as irradiance, temperature and desiccation) that affect their survival/death in the environment [9,10]. Researchers suggest computer-based simulation as a practical method to study microorganisms in stormwater [11].

It is not yet practical to model hundreds of different microorganisms that might be present in the stormwater. Simulation of faecal indicator bacteria (FIB), which is commonly used as indicators of microbiological impairment of water, have been paid much attention. Previous studies have focused mainly on FIB simulations in rivers, lakes or rural runoff [12,13,14,15]. Studies have shown that water catchment quality models, such as the Soil and Water Assessment (SWAT) tool, are not applicable to the simulation of FIBs in urban areas [9]. Some urban NPS models, such as the EPA’s Storm Water Management Model (SWMM), as well as Model of Urban Sewers (MOUSE) and Storage, Treatment, Overflow, Runoff Model (STORM), have been revised to model the FIB contamination in runoff [16]. However, these urban NPS models are originally developed for traditional pollutants and do not represent the mechanisms of survival, mortality and transport of FIBs [17].

McCarthy et al. [9] developed the Micro-Organism Prediction in Urban Stormwater (MOPUS) model for microorganism prediction in urban stormwater. MOPUS is a conceptually based model and has two components including both the surface and subsurface, in which the building-up and washing-off of *E. coli* are calculated. This model obtained reasonably good predictions for *E. coli* contamination. However, MOPUS is spatially lumped and lack the expression of spatial variations on microbial contamination. This will greatly restrict the application of this model for microorganism control in storm water. It is clear that development of a semi-distributed model for predicting faecal coliform in urban stormwater is necessary.

The premise of establishing a semi-distributed model involves obtaining reliable and distributed hydrological simulation results since contamination processes are known to be associated with runoff processes [18]. MOPUS includes both a rainfall runoff model and a microorganism model. These two models can be used together or separately, which provides the possibility of coupling with other hydrological models [9]. However, no published papers have coupled MOPUS with other hydrological models at present. SWMM, which has been used in thousands of sewer and stormwater studies throughout the world, has proved to be a reliable hydrological model [19,20,21]. Coupling SWMM and MOPUS could take full advantage of both the hydrological simulation module of SWMM and microorganism simulation module of MOPUS.

Despite the MOPUS was specifically developed for *E. coli*, it might be applicable for these three FIBs, including faecal coliform (FC), *E. coli* and enterococci, due to the similar processes and mechanisms associated with the variability of intra-event statistics [9,22]. Based on the above theory and assumptions, the main objectives of the present study are: (1) to develop a semi-distributed model for predicting instantaneous FC concentration in urban stormwater systems and (2) to test the performance of the model and complete the parameterization and localization processes.

## 2. Materials and Methods 

### 2.1. Site Description and Data Collection

**Figure 1:**
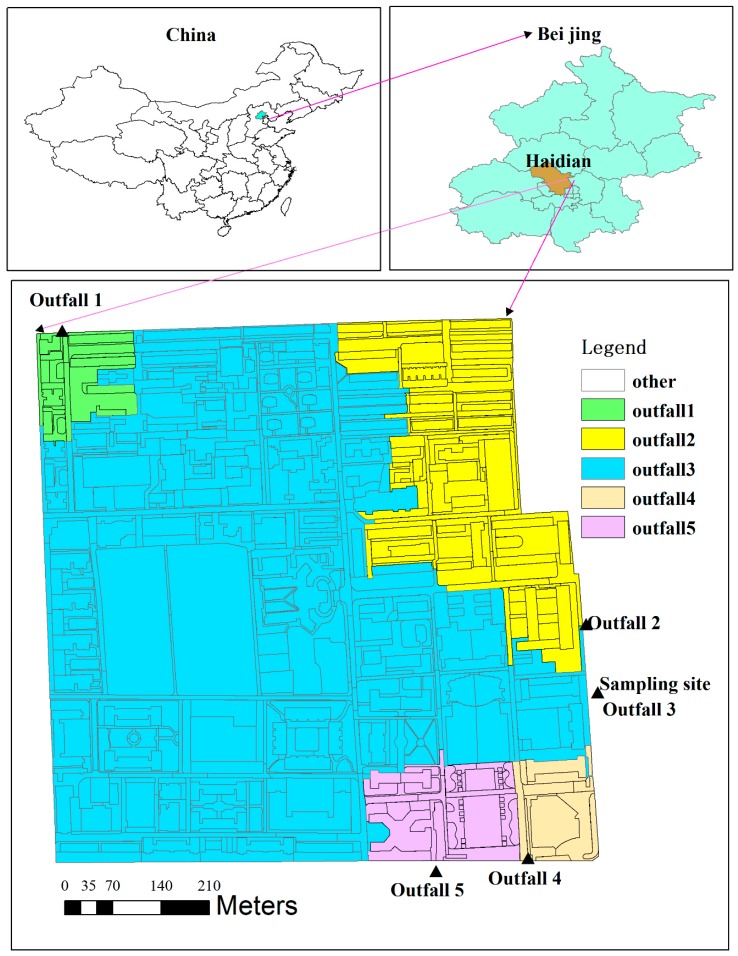
Study area and the locations of sampling site for FC. Green areas-the upstream of outfall 1, yellow areas-the upstream of outfall 2, blue areas-the upstream of outfall 3, orange areas-the upstream of outfall 4, purple areas-the upstream of outfall 5.

As shown in Figure 1, the study catchment is located in the municipality of Beijing (China), between the northern second ring road and third ring road. The catchment occupies an area of 58 ha, of which approximately 30.29% is occupied by buildings and 25.22% is green spaces. The catchment is served by a separate storm sewer system consisting of sewer pipes within a diameter range of 0.1–0.9 m. Runoff generated from the catchment is drained by five outlets to the municipal stormwater pipe network. 

Outfall 3, occupying 73.19% of the total area, was selected for flow and FC monitoring. Once runoff was initiated, water samples were collected manually at outfall 3 at 5-min intervals during the first 30 min of the rainfall event, followed by 10-min intervals for the next 30–60 min and 20-min intervals for the next 60–100 min; subsequently, samples were collected at 30-min intervals until there was no more runoff. A HACH flow meter (FL900, HACH, Loveland, CO, USA) was used to monitor outfall flow in real time, with a time interval of five minutes. A HOBO automated weather station (Onset, Bourne, MA, USA) was installed in the study area to record meteorological data, including atmospheric pressure, temperature, relative humidity, solar radiation, wind speed and rainfall, at five-minute intervals. 

The FC concentrations were measured using the Colilert-18^TM^ method, Defined Substrate Technologies, (IDEXX, Westbrook, ME, USA), following the manufacturer’s instructions. Samples required a 100:1, 1000:1 or 10000:1 dilution due to high counts. Quantification was conducted using a 97-well most probable number (MPN) Quanti-tray/2000. Positive wells were converted to MPN using manufacturer-supplied MPN tables. This method is used to monitor total coliform, FC and *E. coli* in the United States, Europe, and most countries and has been approved by the EPA of the United States [23].

### 2.2. Description of the SWMM and MOPUS Models

#### 2.2.1. SWMM

The EPA’s Storm Water Management Model (SWMM) was first developed in 1971 and has experienced several major upgrades since that time. SWMM version 5.0 has been extended to model green infrastructure practices as low impact development (LID) controls [24]. The latest version 5.1.013 released in 2018 includes a software utility that allows future climate change projections to be incorporated into modeling. SWMM can be used for a single rainfall event or for long-term continuous water quantity and water quality simulation. The core simulation steps of the SWMM model are as follows: ground surface rainfall sub-system calculation; overland flow sub-system calculation and storm sewer flow sub-system calculation [24,25]. There are three options for modelling infiltration in SWMM: the Horton model, the Green-Ampt model, and the SCS Runoff Curve method. In the present study, the Horton model was used to describe the relationship between infiltration rate and time. In the process of infiltration, the infiltration rate increases with time and decreases from maximum to minimum:

(1) $$f_{p}=f_{\infty }+(f_{0}-f_{\infty })e^{-\alpha t}$$

where *f_p_* is the infiltration rate at *t*, mm/s; $f_{\infty }$ is the steady infiltration rate, mm/s; $f_{0}$ is the initial infiltration rate, mm/s; α is the infiltration attenuation coefficient; and *t* is the rainfall time, s.

In a previous study, a hydrologic model with high spatial resolution was established based on the SWMM model [26]. The catchment was delineated according to the sewer network and land use data with high resolution. Field survey was also carried out to confirm the surface and subsurface drainage patterns in order to accurately discretize the subcatchment areas. Among the 749 subcatchments obtained, 728 subcatchments were categorized as single land use. The subcatchment runoff was assigned to the sewer system through the sewer inlet, which was the closest to the subcatchment area centroid. The flow directions between subcatchments were determined using topographical data. Physical catchment characteristics such as catchment area and percentage of impervious area, were derived from land use data. The subcatchment slope was estimated by DEM derived from elevation data of all junctions, and the average slope was 1.7%. The other parameters, such as flow width, Manning’s roughness, Depression storage and Horton’s infiltration parameters were calibrating parameters. Furthermore, the most sensitive parameter in the hydrological simulation, the depth of depression storage in an impervious area (Dstore-imperv), was divided based on the impervious land-use types.

A total of eight independent rainfall events were used for calibration and validation of the hydrologic model. Four events were selected for calibration purposes, and the remaining four rainfall events were used for validation. These rainfalls were representative of the storms occurring in Beijing, ranging from single to multiple peaks and from short to long durations and corresponding to light rain, heavy rain and rainstorm events. The results revealed that the hydrologic model performs very well (NSE range during calibration: 0.854–0.920; NSE range during validation: 0.737–0.912). For more detailed information, please refer to the study by Hou et al [26]. 

#### 2.2.2. MOPUS

The MOPUS model was developed by McCarthy et al [9]. to simulate the *E. coli* levels in stormwater sewer systems using two models: a rainfall runoff model and a microorganism model. The microorganism model includes surface and subsurface components for the simulation of building-up and washing-off from impervious surfaces and sewer pipes. Microorganisms source from animals and humans. Once these microorganisms are deposited, their survival depends on environmental factors, such as temperature, humidity, pH, nutrient level, salinity and toxicity. Then the microorganisms are transported, often by the incidence of runoff. The governing equations of the microorganism model are listed in Table 1. For a detailed introduction to MOPUS, please refer to McCarthy et al [9].

$P_{s}\left(t\right)$ surface storage (orgs), VP(t−1) previous day’s vapor pressure (hpa), $\bar{\mathrm{VP}}$ mean VP(t) value (hpa), RH(t−1) previous day’s maximum relative humidity (%), $\bar{\mathrm{RH}}$ mean RH(t) value (%), PsCoeff, VPCoeff and RHCoeff are the calibration coefficients, $C_{s}\left(t\right)$ Surface wash-off (orgs/L), RI(t) routed and translated rainfall intensity (mm/min), $P_{\mathrm{ss}}\left(t\right)$ subsurface storage (orgs), ${\mathrm{ADWP}}_{v}\left(t\right)$ the number of the days since an event with a rainfall intensity capable of producing velocities in the pipe of V m/s, $P_{\mathrm{ss}}\mathrm{Coeff}$ the magnitude of the wastewater cross connection problem at each site (orgs), $C_{\mathrm{ss}}\left(t\right)$ subsurface wash-off (orgs/L), A the time since the start of the rainfall events.

### 2.3. Development of the MOPUS_S Model

The semi-distributed model developed in the present study was named MOPUS_S. The development from MOPUS to MOPUS_S model mainly include three aspects: considering the effects of land use types on microbial accumulation, coupling SWMM with MOPUS model to make full use of the advantages of hydrological simulation and changing the constant in MOPUS to a calibration parameter in MOPUS_S to complete the localization of parameters. Soft coupling is used in this study. Matlab was used to implement all the algorithms and to provide a platform for input/output data transfers between the SWMM and MOPUS models. The specific algorithms of MOPUS_S were as follows:

(1) Surface storage in a single sub-catchment:

(2) $$P_{\mathrm{si}}\left(t\right)=10^{P_{\mathrm{si}}\mathrm{Coeff}}\times {\left[\frac{\mathrm{VP}(t-1)}{\bar{\mathrm{VP}}}\right]}^{\mathrm{VPCoeff}}\times {\left[\frac{\mathrm{RH}(t-1)}{\bar{\mathrm{RH}}}\right]}^{\mathrm{RHCoeff}}\times S_{i}\;(\mathrm{orgs})$$

where $P_{\mathrm{si}}\left(t\right)$ is the pollutant accumulated in the ith sub-catchment area, orgs; $S_{i}$ is the area of the ith sub-catchment, ha; and $P_{\mathrm{si}}\mathrm{Coeff}$ is the accumulation coefficient, which is classified into the accumulation parameters for roofs, $P_{\mathrm{srf}}\mathrm{Coeff}$, the accumulation parameters for green land, $P_{\mathrm{sg}}\mathrm{Coeff}$, and the accumulation parameters for roads, $P_{\mathrm{sr}}\mathrm{Coeff}$. VP(t−1) is the previous day’s vapor pressure, hpa; $\bar{\mathrm{VP}}\;\mathrm{is}$ the mean VP(t) value, hpa; RH(t−1) is the previous day’s maximum relative humidity, (%); $\bar{\mathrm{RH}}$ is the mean RH(t) value, (%).The vapour pressure was calculated from the wet bulb temperature at 9 a.m., the relative humidity was derived from data obtained from the meteorological station, and $S_{i}$ was extracted from the SWMM output.

Unlike MOPUS, this formula does not calculate the overall surface storage in the entire study area; instead, it obtains the surface storage in each sub-catchment. This process makes it possible to both account for the smallest unit of the SWMM and fully account for the impact of different land-use types.

(2) Surface wash-off in a single sub-catchment:

(3) $$C_{\mathrm{si}}\left(t\right)=\frac{P_{si}\left(t\right)\times {\left[\frac{{6Q}_{i}\left(t\right)}{S_{i}}\right]}^{C_{s}\mathrm{Coeff}}}{{6\times 10}^{5}Q_{i}\left(t\right)}\;(\mathrm{org}/100\;\mathrm{mL})$$

where $Q_{i}\left(t\right)$ is the surface runoff of the *i*th sub-catchment, which was derived from the SWMM output (converted to mL/min), and the constants in the formula are for unit conversion. The final result in orgs/100 mL corresponds to our monitoring measurement of most probable number (MPN)/100 mL. $C_{s}\mathrm{Coeff}$ is the wash-off coefficient.

RI(t) in MOPUS model is the routed and translated rainfall intensity (mm/min), representing the routing and translation of pollutants which occurs in urban catchments [9]. In MOPUS_S, RI(t) is replaced by $Q_{i}\left(t\right)$ in this formula for the usage of hydrological simulation results based on SWMM model and the expression of spatial variations. In MOPUS, The relationship between rainfall drop kinetic energy and rainfall intensity is modelled using a power function (i.e., KE(t) ∝ RI(t)EXP). EXP is a constant with specific regional characteristics. Moreover, RI(t) in MOPUS is replaced by $Q_{i}\left(t\right)$ in Equation (3). Therefore, the constant coefficient (1.293) in MOPUS is replaced by a calibration parameter $C_{s}\mathrm{Coeff}$.

(3) Total surface wash-off:

(4) $$C_{s}\left(t\right)=\sum C_{\mathrm{si}}\left(t\right)\;(\mathrm{org}/100\;\mathrm{mL})$$

where $C_{s}\left(t\right)$ is the total surface wash-off and represents the total microbial biomass of all sub-catchments.

(4) Subsurface storage:

(5) $$P_{\mathrm{ss}}=10^{P_{\mathrm{ss}}\mathrm{Coeff}}\times {\mathrm{ADWP}}_{v}\left(t\right)\;(\mathrm{orgs})$$

where $P_{\mathrm{ss}}$ is the subsurface storage, $P_{\mathrm{ss}}\mathrm{Coeff}$ is the calibration coefficient representing the accumulation capability of the sewer system. ${\mathrm{ADWP}}_{v}$ is the antecedent dry period and the time unit was set to hours to make the description more accurate. Additionally, v is a calibration parameter in MOPUS that represents the velocity of antecedent stormwater events that are capable of resuspending deposited microorganisms. McCarthy et al. [9] found that v was relatively constant for all sites. It is logical since the shear forces (and therefore the velocity of the flow) required to resuspend microorganisms should not be site specific. Therefore, v was fixed at a value of 1 m/s in this study. 

(5) Subsurface wash-off:

(6) $$C_{\mathrm{ss}}\left(t\right)=P_{\mathrm{ss}}\times Q_{0}\left(t\right)\times {\left[\sum_{j=A}^{t}Q_{0}\left(j\right)+0.1\right]}^{-1}\times 10^{-1}\;\mathrm{org}/100\;\mathrm{mL})$$

where $Q_{0}\left(t\right)$ is the flow of the outfall 3, (m3/s), and A is the beginning of the rainfall. Similar to MOPUS, the subsurface component simulates the study area as a whole. $Q_{0}\left(t\right)$, derived from the SWMM simulation results, is used here to replace RI(t) in MOPUS.

(6) Total wash-off: The total wash-off of microorganisms equals the summation of the surface and subsurface wash-off.

(7) $$C\left(t\right)=C_{s}\left(t\right)+C_{\mathrm{ss}}\left(t\right)\;(\mathrm{org}/100\;\mathrm{mL})$$

### 2.4. Calibration and Validation

Since automatic sampling equipment is rarely used in China, runoff sampling can only be carried out manually. The rainfall in the study area is concentrated in the rainy season, which is sudden and mostly occurs at night, so it is difficult to capture many effective rainfall events. Therefore, two rainfall events (rainfall 0804 and 0830) were used for calibration and two events (rainfall 0729 and 0831) were used for validation of the MOPUS_S model. There are seven calibration parameters related to MOPUS_S: $P_{\mathrm{srf}}{\mathrm{Coeff},\;P}_{\mathrm{sg}}{\mathrm{Coeff},\;P}_{\mathrm{sr}}{\mathrm{Coeff},\;\mathrm{VPCoeff},\;\mathrm{RHCoeff},\;C}_{s}\mathrm{Coeff}$ and $P_{\mathrm{ss}}\mathrm{Coeff}.$ The genetic algorithm (GA) toolbox of Matlab2012 was applied for automatic parameterization. The objective function ∅, which need to be minimized, was as follows:

(8) $$\emptyset =\frac{{\sum_{l=1}^{L}(M_{l}-P_{l})}^{2}}{\sum_{l=1}^{L}{\left(P_{l}-\bar{P}\right)}^{2}}$$

where L is the number of observations and $M_{l}$ and $P_{l}$ represent the simulated and observed values, respectively, $\bar{P}$ is the observed mean values. 

The number being calibrated in this study is relatively high, which might cause equifinality (multiple models are all acceptable to represent a system) [27]. A repeated approach similar to a Monte Carlo approach was adopted. Parameter calibration through GA method was conducted in two steps. In the first step, the calibration range was determined by combination of the parameter range in MOPUS and the range of monitored microbial concentrations in our study. The parameter sets were chosen based on their performance ranking at the first step (i.e., the smaller the value of ∅ is, the better the model performance is). The parameter distributions were compared to determine the sensitivity of the model to each parameter. For example, a wide, flat distribution indicated that the model outputs were not very sensitive to changes in the parameter, whilst a narrow, sharp distribution indicated that the model outputs were sensitive to the parameter’s value [28]. In the second step, the calibration ranges of the parameters were adjusted based on the frequency distribution in the first step. The search process was terminated when the objective function values could not be significantly improved in five iteration steps. Two indexes, the Nash-Sutcliffe efficiency (NSE) and correlation index (R^2^) were used to evaluate the parameter calibration and validation. 

## 3. Results

### 3.1. Contamination Characteristics of FC in the Stormwater Pipeline

FC concentration ranged from 6.24 × 10^3^ to 1.99 × 10^6^ MPN/100 mL.The mean concentrations of FC across rainfall events ranged between 1.58 × 10^5^ and 6.31 × 10^5^ MPN/100 mL (Table 2). All of the water samples exceeded the Class V guideline of FC (4 × 10^3^ PN/l00mL) according to the Chinese national surface water environmental quality standard GB3838-2002 [29]. FC in the sewer pipes not only originated from the wash off from the underlying surfaces but also came from the existed accumulation in the sewer pipe [9]. Moreover, the combination of moisture, anaerobic conditions and the lack of sunlight make this environment very conducive for microorganism growth [9,30]. Especially, large amounts of sediment inside the stormwater pipe can be a serious issue, as it does not necessarily affect the delivery efficiency of the pipe, so the problem can be more widespread than estimated [31]. This is why the microorganisms inside the pipe network cannot be ignored.

FC contamination varies largely between different urban catchments due to variable degree of the development, land use type, warm-blooded animal manure, and drainage system characteristics [32]. The maximum FC concentration in the present study area was 1.99 × 10^6^, higher than that of United States, Sweden and Malaysia (4 to 5 orders of magnitude), but lower than that of India (5 to 7 orders of magnitude) [22,32,33,34]. 

**Figure 2:**
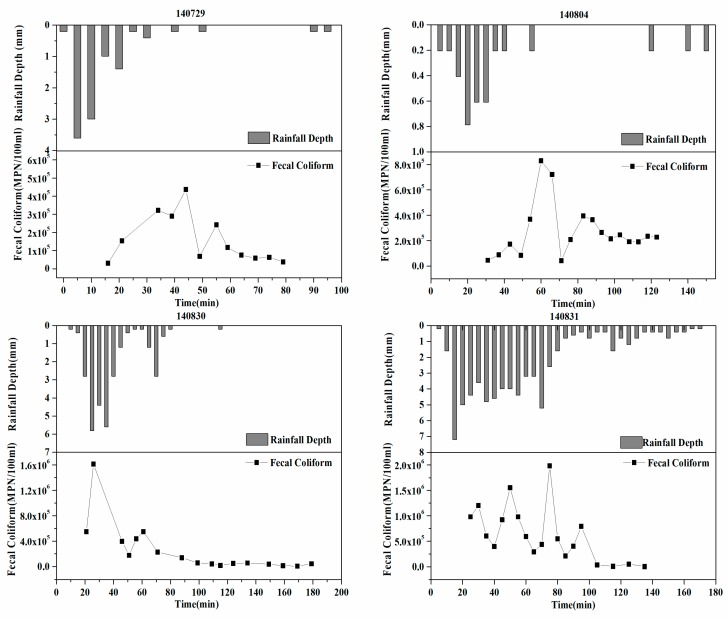
Time distribution of instantaneous concentration of FC in the sewer outlet.

Figure 2. shows the temporal variation in the FC instantaneous concentration. The variability was very large during the rainfall period, and the maximum and minimum values differed by several orders of magnitude. McCarthy et al. [9] proposed a hypothesis for the accumulation and wash-off processes of surface microorganisms: the accumulation of microorganisms during dry periods is much greater than the wash-off during rainfall events. The results of the present study are consistent with this hypothesis. No consistent decrease in pollutant concentration at the end of rainfall was evident, and at the end of a rainfall event, the concentration remained at a high level, with an order of magnitude between 10^3^ and 10^4^.

### 3.2. Parameter Refinement and Localization

**Figure 3:**
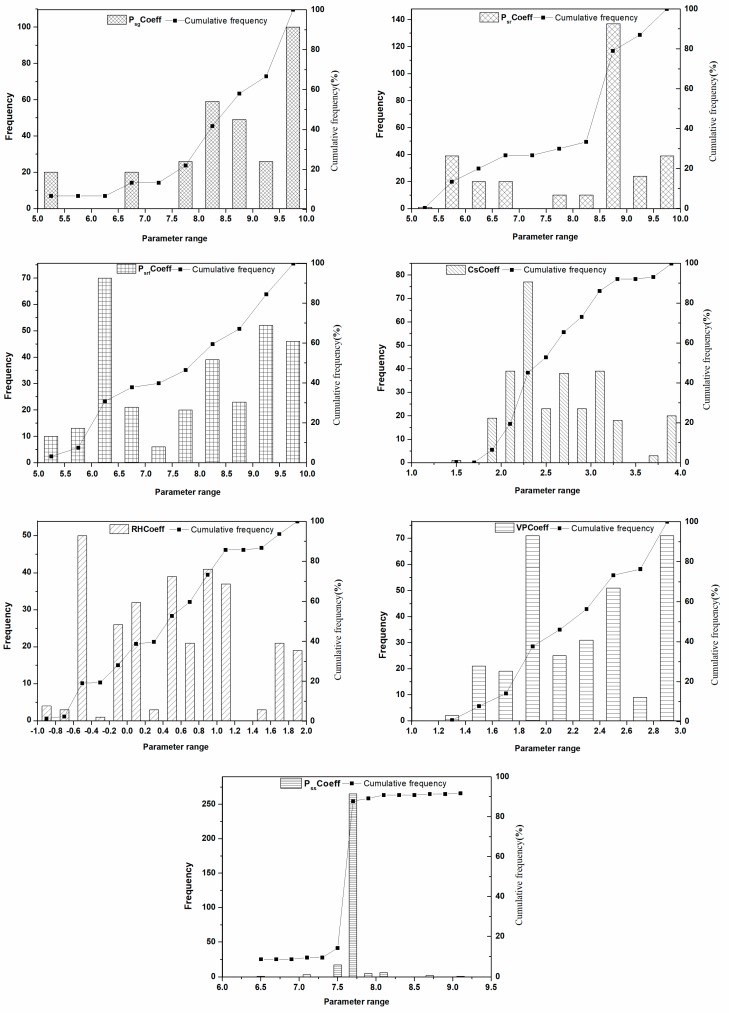
Frequency distribution and cumulative frequency of top ranking parameters in first calibration step.

The parameter sets were ranked based on their performance in the first step (i.e., the smaller the value of ∅ is, the better the model performance is). The frequency distribution of the top ranked parameters (top 300) was analysed to determine the parameter sensitivity. McCarthy et al. [28] found that MOPUS’s outputs were very sensitive to the change in both $P_{s}\mathrm{Coeff}$ and $P_{\mathrm{ss}}\mathrm{Coeff}$. These parameters represent the surface, and subsurface, deposition rates of microorganisms, respectively. As is shown in Figure 3, the sharp, narrow distribution found for $P_{\mathrm{ss}}\mathrm{Coeff}$ indicate that the MOPUS_S’s outputs were also quite sensitive to the change in $P_{\mathrm{ss}}\mathrm{Coeff}$. However, the sensitivity of the parameters related to the surface deposition rates of microorganisms, including $P_{\mathrm{srf}}{\mathrm{Coeff},\;P}_{\mathrm{sg}}\mathrm{Coeff}$ and $P_{\mathrm{sr}}\mathrm{Coeff},$ varied largely in our study. The sensitivity of the model output to the changes in $P_{\mathrm{sg}}\mathrm{Coeff}$ and $P_{\mathrm{sr}}\mathrm{Coeff}$ was higher than that seen for $P_{\mathrm{srf}}\mathrm{Coeff}$. This result indicated the importance of considering the impact of land use on surface deposition rates of microorganisms. $C_{s}\mathrm{Coeff}$, which represents the relationship between the total kinetic energy and runoff during the wash-off process of surface microorganisms, was also sensitive parameter in MOPUS_S. The parameters associated with the microorganism survival processes, including RHCoeff and VPCoeff, were identified as the least sensitive parameters in MOPUS [28]. In this study, the flatter, wider distributions seen in Figure 3 for both RHCoeff and VPCoeff suggest that MOPUS_S’s outputs were also least sensitive to these parameters. 

The calibration ranges of the parameters in the second step were also adjusted according to the frequency distribution of the top ranked parameters. The value of $P_{\mathrm{ss}}\mathrm{Coeff}$ should be within a very narrow range to adequately calibrate the model. Therefore, the calibrated range of $P_{\mathrm{ss}}\mathrm{Coeff}$ was adjusted from 3.0–10.0 in the first step to 6.5–9.5 in the second step (Table 3). The values of accumulation parameters with good performance, $P_{\mathrm{srf}}{\mathrm{Coeff},\;P}_{\mathrm{sg}}\mathrm{Coeff}$ and $P_{\mathrm{sr}}\mathrm{Coeff}$ were within the range of 5–10. Therefore, the calibration ranges of these parameters remained unchanged. The parameters related to death/survival, VPCoeff and RHCoeff, and the wash-off coefficient $C_{s}\mathrm{Coeff}$ were also adjusted slightly.

Optimised parameter values for MOPUS_S are shown in Table 3. Although the equations in MOPUS were modified, the basic theory of the modelling remained consistent. Therefore, in terms of the same parameters, there is still some comparability. The accumulation coefficient $P_{s}\mathrm{Coeff}$ represents the deposition rate of microorganisms on catchment surfaces, and a high $P_{s}\mathrm{Coeff}$ value corresponds to high levels of microorganisms [9]. The calibrated values of $P_{\mathrm{srf}}\mathrm{Coeff},$ $P_{\mathrm{sg}}\mathrm{Coeff}$ and $P_{\mathrm{sr}}\mathrm{Coeff}$ in MOPUS_S were 6.4299, 8.9866 and 8.8289, separately, higher than those in MOPUS model (2.95–4.71 for different sites). It was concluded that the accumulation rate of microorganisms on the underlying surface may be relatively higher in this study area. It should be noted that the accumulation coefficients varied largely for different land use types, representing the different deposition rate of microorganisms. The accumulation coefficient for green areas ($P_{\mathrm{sg}}\mathrm{Coeff}$) and roads ($P_{\mathrm{sr}}\mathrm{Coeff}$) were similar, but the accumulation coefficient for roofs ($P_{\mathrm{srf}}\mathrm{Coeff}$) was relatively lower.

McCarthy et al. [28] found that vapour pressure and relative humidity can explain the variations in *E. coli* measurements in the watershed between events and used the two variable parameters, VPCoeff and RHCoeff, to model microbial survival/death rate. The VPCoeff in MOPUS was always positive. For different sites, however, the RHCoeff values were positive or negative. In our study, the optimised values of VPCoeff and RHCoeff were 2.4266 and −0.5259, respectively, which was consistent with McCarthy’s results. 

In MOPUS, the relationship between raindrop kinetic energy and rainfall intensity was modelled using a power function, and the exponent was fixed at 1.293. However, this value can vary among different regions and rainfall conditions. In MOPUS_S, $C_{s}\mathrm{Coeff}$ was used to characterize the relationship between the total kinetic energy and runoff during the wash-off process of surface microorganisms in our study area, and the value was 2.8280.

$P_{\mathrm{ss}}\mathrm{Coeff}$ accounts for a variety of different subsurface mechanisms and mainly represents the variability in the number and severity of illegal wastewater intrusions into the stormwater system [9]. The value of $P_{\mathrm{ss}}\mathrm{Coeff}$ was 6.599 in our study, much higher than that of $P_{\mathrm{ss}}\mathrm{Coeff}$ in the MOPUS model (ranging from 0.96 to 1.39). This result revealed the high pollution state of microorganisms in the stormwater pipe network in the present study area. Although the study catchment is served by a separate storm sewer system, the presence of illicit connection between storm and sewage sewers was also found through the network data. Moreover, the old sewer networks, sediment inside and inadequate drainage capacity might also have contributed the high level of microorganism storage in the drainage system.

### 3.3. Performance of MOPUS_S

**Figure 4:**
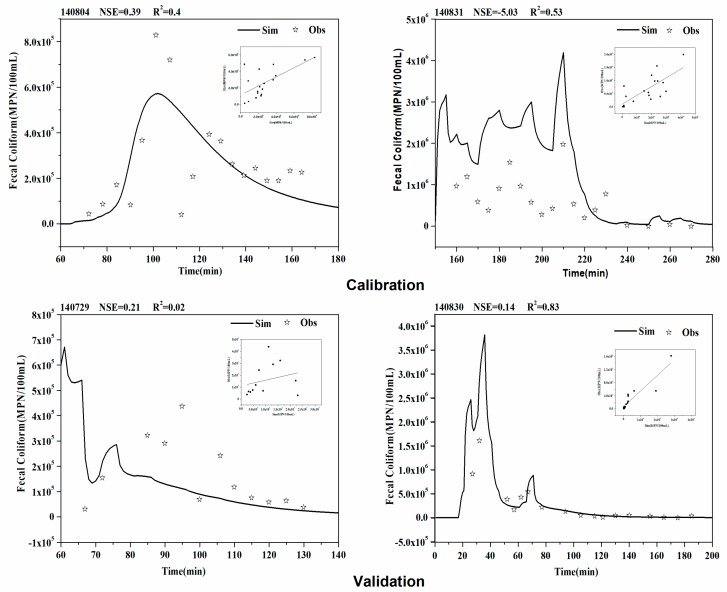
Calibration and validation results for MOPUS_S.

Figure 4 presents the typical measured and predicted pollutogarphs and corresponding evaluation indexes in the calibration and validation processes. Generally, the simulated trend is consistent with the measured trend. The calibrated model yielded an efficiency of NSE ranging from 0.14 to 0.39 and R^2^ ranging from 0.4 to 0.83. In the validation process, the values of NSE were 0.21and −5.3 for rainfall 140729 and rainfall 140831, respectively. The performance varied largely among rainfall events, in accordance with the results of MOPUS. McCarthy et al. [9] obtained similar results of the simulation performance of MOPUS, with NSE values in the range of −25.1 to 0.79 for different rainfall events. The performance for the rainfall 140831 was lower than the other three events, possibly due to the short duration but continuous heavy rain type, which are quite different from the other three events. Therefore, the calibrated parameter values did not fit well enough with the simulation of this rainfall type.

MOPUS_S well predicts low and medium concentrations but shows limitation in predicting peak concentrations, which obviously affect the value of evaluation indexes. Cho et al. [35] used a modified SWAT model for simulating FC in the Wachusett Reservoir Watershed and obtained the similar results. Higher uncertainties associated with measurement at peak concentrations might cause high modelling errors. For the Colilert monitoring method, the samples needed to be diluted 100 or 1000 times to be within the monitoring range (0–2,419.60 MPN/100 mL) [36]. For samples with high concentrations, higher dilution ratio increases the uncertainty in the concentration measurements. 

There are few reports on microbial simulations in urban stormwater. The previous studies regarding microbial simulations focused mainly on rural rivers and estuaries [37,38,39]. Comparisons could only be made to the rural based model and MOPUS (Table 4). The previous studies applying SWAT model to FC simulation show NSE values ranged from −6.0 to 0.52. Coffey et al. [37] used SWAT to simulate *E. coli* in rural catchments and the NSE ranged between −0.42 to 0.29. Cho et al. [35] simulated the FC concentrations in a reservoir basin with the improved SWAT model and the NSE values ranged from −0.28 to −0.18. Thus, compared to MOPUS and other existing bacterial models, MOPUS-S in the present study clearly achieved reasonable simulation results for the single rainfall event. 

It should be noted that modeling bacteria still faces many difficulties, and the simulation performance is poorer to a certain degree than that of physico-chemical pollutants. One of the possible reasons is that the uncertainties in microbial monitoring, due in part to sample collection, preservation/storage, and laboratory analysis, are greater than those in sediment and nutrient analysis [40]. For example, when using Colilert technology to monitor *E. coli*, the average analysis uncertainty is greater than 27% [41]. For microbial data at a single sampling point, the uncertainty is greater than 30%, even up to 67% [30]. Model structure inadequacies could also be an important reason. Unlike physico-chemical pollutants, microbial contamination is not only related to the building-up and washing-off of the accumulated microorganisms, but it is also dependent on the factors affecting survival and death in the environment [9,10].

## 4. Conclusions

In this study, MOPUS_S, a semi-distributed model for predicting faecal coliform in separate drainage systems, was developed by integrating SWMM and MOPUS models. MOPUS_S could take full advantage of both the hydrological simulation module of SWMM and microorganism simulation module of MOPUS. The main conclusions are summarized below:

(1) MOPUS_S’s outputs were quite sensitive to the change in $P_{\mathrm{ss}}\mathrm{Coeff}$ and $C_{s}\mathrm{Coeff}$. The sensitivity of the accumulation parameters for different land uses varied largely. The parameters associated with the microorganism survival processes, including RHCoeff and VPCoeff, were identified as the least sensitive parameters.
(2) The MOPUS_S model showed comparable performance to that of MOPUS and other rural based bacterial models. Considering substantial uncertainty and insufficient information related to bacterial modelling, the simulation performance is acceptable.
(3) The model performance varied largely between rainfall events and showed better performance if the low and medium FC concentrations were obtained. This emphasizes the significant impact of measurement uncertainty related to peak concentrations.

There are several factors should be considered when applying MOPUS_S at this stage. Firstly, the number of rainfall events for calibration and validation is low, which might skew the reported parameter values. Therefore, caution should be taken in the application of reported parameters. Secondly, more rainfall events with different rainfall types should be used for calibration and validation, considering the model performance varied largely between rainfall events. Thirdly, the uncertainty related to measuring the peak concentrations should be reduced as far as possible. Further development of MOPUS_S will depend on comprehensive factors, including numerous and continuous monitoring, more understanding of the contaminant mechanisms and improvement of model structure.

## Figures and Tables

**Table 1:** The governing equations of the microorganism model in MOPUS.

Model Equation	Comment
$P_{s}\left(t\right)=10^{\mathrm{PsCoeff}}\times {\left[\frac{\mathrm{VP}(t-1)}{\bar{\mathrm{VP}}}\right]}^{\mathrm{VPCoeff}}\times {\left[\frac{\mathrm{RH}(t-1)}{\bar{\mathrm{RH}}}\right]}^{\mathrm{RHCoeff}}$	Surface storage
$C_{s}\left(t\right)=\frac{P_{s}{\left(t)\times \mathrm{RI}(t\right)}^{1.293}}{\mathrm{RI}(t)}$	Surface wash-off
$P_{\mathrm{ss}}\left(t\right)=10^{P_{\mathrm{ss}}\mathrm{Coeff}}{\times \mathrm{ADWP}}_{v}\left(t\right)$	Subsurface storage
$C_{\mathrm{ss}}\left(t\right)=P_{\mathrm{ss}}\times \mathrm{RI}(t)\times {\left[\overset{t}{\underset{i=A}{\sum }}\mathrm{RI}\left(i\right)+0.1\right]}^{-1}$	Subsurface wash-off

**Table 2:** Summary of statistics of FC concentrations.

Rainfall Events	Max	Min	Mean	St. Dev.
2014/07/29	4.37 × 10^5^	3.08 × 10^4^	1.58 × 10^5^	1.33 × 10^5^
2014/08/04	8.30 × 10^5^	4.22 × 10^4^	2.74 × 10^5^	2.17 × 10^5^
2014/08/30	1.62 × 10^6^	8.6 × 10^3^	2.82 × 10^5^	4.23 × 10^5^
2014/08/31	1.99 × 10^6^	6.24 × 10^3^	6.31 × 10^5^	5.42 × 10^5^

**Table 3:** Parameters calibration range and the value of calibrated parameters.

Parameters	Cumulative Coefficient			Survival/Death Coefficient		Washing-Off Coefficient	Underground Cumulative Coefficient
	$P_{\mathrm{srf}}\mathrm{Coeff}$	$P_{\mathrm{sg}}\mathrm{Coeff}$	$P_{\mathrm{sr}}\mathrm{Coeff}$	VPCoeff	RHCoeff	$C_{s}\mathrm{Coeff}$	$P_{\mathrm{ss}}\mathrm{Coeff}$
Calibration range in the first step	5–10	5–10	5–10	1–3	−3–2	1–4	3–10
Calibration range in the second step	5–10	5–10	5–10	1.2–3	−1–2	1.5–4	6.5–9.5
Calibrated value	6.4299	8.9866	8.8289	2.4462	−0.5259	2.8280	6.5990
Parameters in MOPUS	$P_{s}\mathrm{Coeff}$			VPCoeff	RHCoeff	—	$P_{\mathrm{ss}}\mathrm{Coeff}$
Calibrated value	2.95~4.71			3.01~5.01	−3.4~1.4	—	0.96~1.39

**Table 4:** Modelling efficiency of microorganisms.

Study Area	Microbial Type	NSE for Single Event	Models	References
Melbourne, Australia	*E. coli*	−25.1–0.79	MOPUS	[9]
Kilshanvey, Ireland	*E. coli*	−0.42–0.29	SWAT	[37]
James River, Missouri, USA	*E. coli*	−6.0–0.21	SWAT	[38]
Rock Creek, Kansas, USA	*E. coli*	−2.2–0.52	SWAT	[39]
Wachusett Reservoir Watershed, Massachusetts, USA	FC	−0.28–0.18	SWAT	[35]
Beijing, China	FC	−5.03–0.39	MOPUS_S	The present study
